# Advancing the understanding of Autism Spectrum Disorders Through Integrative Genomic, Phenotypic and Computational Approaches

**DOI:** 10.1192/j.eurpsy.2026.10739

**Published:** 2026-07-31

**Authors:** N. Bouayed Abdelmoula, B. Abdelmoula

**Affiliations:** LR23ES07 Genomics of Signalopathies at the service of Precision Medicine, Medical University of Sfax, Sfax, Tunisia

## Abstract

**Introduction:**

Autism spectrum disorders (ASD) are genetically and clinically heterogeneous, which complicates diagnosis and treatment. Recent genomic research aims to clarify the complex genetic architecture of ASD to enable precision medicine approaches.

**Objectives:**

This literature review summarizes the results of recent large-cohort genomic studies, whole genome sequencing (WGS), gene discovery efforts and computer analyses published between 2023 and 2025, with an emphasis on the usefulness of genetic tests, subtype classification and integrative genomic approaches.

**Methods:**

We conducted a comprehensive review of the scientific literature using the following keywords: Precision medicine, Autism, Genomics.

**Results:**

Major advances in autism genomics research include the identification of more than 230 new genes and the recognition of distinct autism subtypes. In fact, in a large study analyzing over than 5,000 autistic children, an integrative approach of extensive behavioral traits with genetic data using advanced computational models identifies four subtypes of autism. While the “social and behavioral” and “moderate challenge” groups, reach the developmental stages on time and differ mainly in the severity of the symptoms or in the concomitant conditions, the “mixed challenges” and “broadly affected” groups, face delays and more complex features. One subtype shows high rates of de novo mutations affecting prenatal brain development, while another with predominant social and behavioral challenges involves genes active postnatally, suggesting diverse biological timelines for autism manifestation. This subtype classification marks a paradigm shift toward precision medicine in autism by enabling more accurate diagnosis and personalized care based on genetic and clinical subtypes. Overall, the results reflect a transition from large-scale gene discovery to integrative genomic, phenotypic and computational approaches that allow a subtype-specific understanding of autism, supporting early diagnosis, familial counselling, personalized treatments and better clinical outcomes. These advances lays the foundations for precision psychiatry approaches adapted to various ASD profiles.

**Conclusions:**

These results highlight the genetic and clinical diversity of autism spectrum disorders, the efficiency of WGS compared to other genetic tests, as well as the power of the integration of AI and explainable computer models to improve the understanding of the heterogeneity of ASD. Other new findings, include common co-occurring conditions: epilepsy and intellectual disability as well as autism and myotonic dystrophy 1 du to disrupted gene splicing that affects brain function and social behavior.

**Disclosure of Interest:**

None Declared

